# Long Non-Coding RNA Deregulation in Tongue Squamous Cell Carcinoma

**DOI:** 10.1155/2014/405860

**Published:** 2014-06-22

**Authors:** Wei Gao, Jimmy Yu-Wai Chan, Thian-Sze Wong

**Affiliations:** Department of Surgery, LKS Faculty of Medicine, The University of Hong Kong, Queen Mary Hospital, 102 Pokfulam Road, Kowloon, Hong Kong

## Abstract

*Background.* The deregulated tumorigenic long non-coding RNA (lncRNA) has been reported in several malignancies. However, there is still no comprehensive study on tongue squamous cell carcinoma (SCC). *Methods.* Functional reannotation for the human lncRNA was carried out by ncFANs. Real-time quantitative PCR was used to validate the identified lncRNAs. *Results.* Using the functional annotation algorithm from ncFANs, 8 differentially expressed lncRNAs were identified. Lnc-PPP2R4-5, lnc-SPRR2D-1, lnc-MAN1A2-1, lnc-FAM46A-1, lnc-MBL2-4:1, and lnc-MBL2-4:3 were upregulated in the microdissected tongue SCC tissues. In comparison, lnc-AL355149.1-1 and lnc-STXBP5-1 showed significant downregulation. High level of lnc-MBL2-4:3 was significantly associated with the node positive tongue SCC patients. Further, patients with advanced T-stage demonstrated a further reduction of lnc-AL355149.1-1 in the tumor tissues. Treatment of tongue SCC cells with 5-fluorouracil and paclitaxel can reserve the expression patterns observed in the tongue SCC tissues. Further, changes of lnc-MBL2-4:3 and lnc-AL355149.1-1 expression levels were noticed in the cisplatin-resistant tongue SCC cells. *Conclusions.* Our results demonstrated that functional reannotation allows us to identify novel lncRNAs using the existing gene expression array dataset. The association of lncRNA with the T-stage and nodal status of tongue SCC patients suggested that lncRNA deregulation was involved in the pathogenesis of tongue SCC.

## 1. Introduction

Tongue squamous cell carcinoma (SCC) is a common epithelial cancer identified in the oral cavity. In USA, tongue is the most common site of oropharyngeal cancer (in comparison with mouth, pharynx, and others in oral cavity) and the major histological form is squamous cell carcinoma [1]. Major causative factors included tobacco consumption and alcohol abuse. Viral infection including human papilloma virus and Epstein-Barr virus infection might also play a part in the development of tongue SCC [2]. Tongue SCC is an aggressive tumor with rapid growth rate and high chance of regional and distant metastasis. Tumor dimension and the existence of extracapsular spread (ECS) are predictors of survival [3]. In addition, regional spreading to the cervical lymph node and distant metastasis of tongue SCC are indicators of poor prognosis [4, 5].

Different from the mRNA transcript, the codon on the long non-coding RNA (lncRNA) do not code for any peptides or proteins. lncRNA refers to RNA molecules with size over 200 b.p. long and without protein coding functions. To date, the precise control mechanisms of lncRNA are not completely understood. LncRNA could be transcribed by RNA polymerases I, II, and III [6]. LncRNA functions as epigenetic regulators in the somatic cells and is directly involved in cell cycle regulation and cell differentiation [7]. Although the precise regulatory mechanism of lncRNA is not fully understood, evidence suggested that expression of certain lncRNA is being modulated by external stimulus, such as cellular irradiation and chemotherapeutic agents treatment [7, 8]. Further, lncRNA is alleged to be involved in the development of resistant phenotype and deters the efficacy of cancer treatment [9].

To the best of our knowledge, comprehensive lncRNA study aiming at identifying novel lncRNA signature has not yet been carried out in the tongue squamous cell carcinoma. Hence, in the present study, we aimed at identifying the candidate lncRNA associated with tongue SCC using the microarray dataset available in the public microarray data repository. Real-time quantitative PCR was then used to confirm the expression and validate the results in the primary tongue SCC tissues and the paired normal epithelia. We also correlated the expression patterns with the clinical characteristics of tongue SCC patients in order to reveal any potential clinical use of the lncRNA.

## 2. Materials and Methods

### 2.1. Microarray and Functional Reannotation

Microarray data set GSE9844 containing 38 microarray data (http://www.ncbi.nlm.nih.gov/geo/query/acc.cgi?acc=GSE9844/) was obtained from Gene Expression Omnibus (GEO). The dataset contains 26 microdissected tongue squamous cell carcinoma tissues and 12 control tissues examined with Human Genome U133 (HG-U133) Plus 2.0 array (Affymetrix).

### 2.2. Functional Reannotation

The probes on the HG-U133 Plus 2.0 array were reannotated for human lncRNA using non-coding RNA function annotation server (ncFANs) as described [10]. Differential expressed lncRNAs were selected using Student's* t*-test. A *P* value below 0.05 was considered as differentially expressed lncRNAs. The *P* value of differential expression was adjusted with the Benjamini and Hochberg correction for multiple comparisons.

### 2.3. Patient Samples

Tongue SCC patients were recruited at Department of Surgery, The University of Hong Kong, Queen Mary Hospital, Hong Kong. Written consent of tissue donation for research purposes was obtained from patients before tissue collection. The protocol was approved by the IRB of the hospital (IRB reference number UW 12-123). Paired tumor and normal tissues (*n* = 32) were collected. Histological confirmation of the tissues was performed by hospital's pathologists.

### 2.4. Real-Time Quantitative PCR

Total RNA was extracted and purified from the frozen tissues using TRIzol reagent (Invitrogen). The quality and quantity of the isolated RNA were examined with Nanodrop. Reverse transcription was carried out using cDNA conversion kit (Invitrogen). Primers for lncRNA detection and quantification were designed at Universal ProbeLibrary Assay Design Center (http://www.roche-applied-science.com/). The LNA-labeled probe was obtained from the Universal Probe Library (Roche Applied Science). The lncRNA transcript levels were measured by LightCycler 480 (Roche Applied Science) and normalized with the* GAPDH* levels using 2^−ΔCt^ algorithm. Primer and probe sequences were listed in Table 1.

### 2.5. Cell Culture and Drug Treatment

Tongue SCC cell line HN21B was cultured in RPMI 1640 medium supplied with 10% fetal bovine serum (Gibco), 200 Unit/mL penicillin G sodium (Gibco), 200 *μ*g/mL streptomycin Sulfate (Gibco), and 0.5 *μ*g/mL amphotericin B (Gibco). The cell line was incubated in humidified incubator with 5% CO_2_ at 37°C. 5-Fluorouracil (5-Fu) and paclitaxel were obtained from sigma. Stock solutions were prepared by dissolving the drugs in dimethyl sulfoxide (DMSO) at the concentration of 100 mM and 1.2 mM respectively. Cisplatin (Sigma) was dissolved in distilled water at a concentration of 3.3 mM. The stock solutions were freshly diluted to appropriate concentrations in RPMI 1640 medium before treatment. HN21B cells were treated by 0–10 *μ*M 5-Fu, 0–8 nM paclitaxel, or 0–64 *μ*M cisplatin for 72 hours followed by in vitro toxicity test. The toxicity test was performed using the Toxicology Assay Kit Sulforhodamine B (SRB) assay (Sigma-Aldrich) according to manufacturer's manual. The inhibitory concentration (IC) was calculated from the dose-response curve of HN21B upon drug treatment.

### 2.6. Development of Cisplatin-Resistant HN21B Cell Line

The cisplatin-resistant HN21B cell line was developed by chronic cisplatin treatment. HN21B cells were exposed to cisplatin for 3 days, followed by growth recovery in drug-free medium. The concentration of cisplatin was increased in the subsequent cycle and the procedure was repeated until resistance was achieved in HN21B cells.

### 2.7. Immunocytochemistry

HN21B cells were seeded on glass slides and fixed with 4% paraformaldehyde. The nucleus was stained by blue-fluorescent 4′,6-diamidino-2-phenylindole (DAPI) (Invitrogen); F-actin was labeled in red with Alexa Fluor 635 phalloidin (Invitrogen).

### 2.8. Statistical Analysis

Statistical analysis was performed using SPSS V16.0 (SPSS, Chicago, IL). The statistical difference between tongue SCC and paired normal epithelia was examined using Wilcoxon signed-rank test. All the tests were two-sided. *P* value <0.05 was considered as statistically significant.

## 3. Results 

### 3.1. Differential Expressed lncRNA in Tongue Squamous Cell Carcinoma

Using the functional annotation algorithm from ncFANs, 8 differentially expressed lncRNAs were identified (Table 2). Of the 8 lncRNA, lnc-PPP2R4-5, lnc-SPRR2D-1, lnc-MAN1A2-1, lnc-FAM46A-1, lnc-MBL2-4:1, and lnc-MBL2-4:3 were upregulated in the microdissected tongue SCC tissues. In comparison, lnc-AL355149.1-1 and lnc-STXBP5-1 showed significant downregulation in the tongue SCC.

### 3.2. Validation of lncRNA by Real-Time Quantitative PCR

Of the 8 lncRNAs, lnc-MAN1A2-1 (OTTHUMG00000012147) was not detectable in all tissue samples. Lnc-MBL2-4:1 (OTTHUMT00000048111) was detectable in 3% (1/32) tumor tissue samples and was not detectable in all the normal tissue samples. Lnc-FAM46A-1 (OTTHUMG00000015099) was detectable in 22% (7/32) of tumor tissue samples and 13% of (4/32) normal tissue samples.

**Figure 1:**
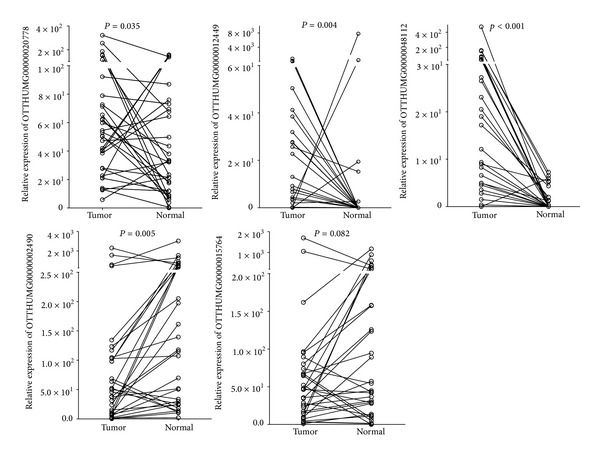
Relative expression levels of lncRNAs in paired tumor and normal tissue samples from patients with tongue SCC. The relative expression levels were normalized to* GAPDH* by qPCR analysis and the data are displayed as 2^−ΔCt^. The difference between tumor and normal was calculated using the Wilcoxon signed-rank test.

For the remaining 5 lncRNAs, 3 were found to be significantly upregulated in the primary tongue SCC tissues. Overexpression of lnc-SPRR2D-1 (OTTHUMG00000012449) and lnc-PPP2R4-5 (OTTHUMG00000020778) was found in the tongue SCC tissue (*P* = 0.004 & 0.035 resp., Wilcoxon signed-rank test). Overexpression of the lnc-MBL2-4:3 (OTTHUMT00000048112) in the tongue SCC was highly significant in comparison with the paired normal epithelia (*P* < 0.001, Wilcoxon Signed Ranked test). Lnc-AL355149.1-1 (OTTHUMG00000002490) was the only lncRNA found to be downregulated in the tongue SCC tissues (*P* = 0.005, Wilcoxon signed-rank test). The fold-change patterns (upregulated/downregulated) in the tongue tissues matched the fold-change patterns in the microarray dataset (Figure 1).

### 3.3. Correlations with Clinicopathological Parameters of Tongue SCC Patients

**Figure 2:**
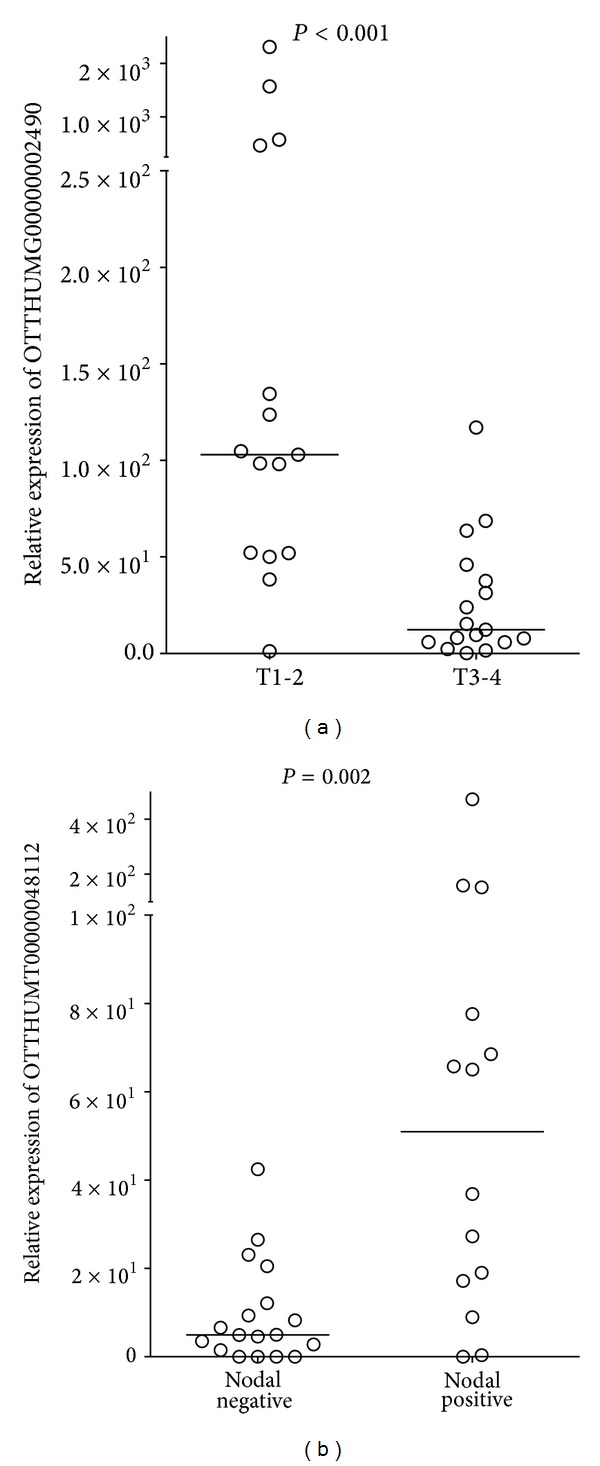
Relative expression levels of lncRNAs in tongue SCC patients with different T-stage (a) and nodal status (b). The relative expression levels were normalized to* GAPDH* by qPCR analysis. The difference was calculated using the Mann-Whitney *U* test.

The expression levels of the 4 differentially expressed lncRNAs were correlated with the clinical parameters of the patients (Table 3). High level of lnc-MBL2-4:3 (OTTHUMT00000048112) was significantly associated with the node positive tongue cancer patients compared to the node-negative patients (*P* = 0.002, Mann-Whitney* U* test). Further, lnc-AL355149.1-1 (OTTHUMG00000002490), the downregulated lncRNA in tongue SCC tissues, displayed substantial downregulation when the cancer progressed from early (T1-2) to advanced stages (T3-4).  Figure 2 showed the expression difference of lnc-MBL2-4:3 and lnc-AL355149.1-1 in the tongue SCC tissues according to the T-stage and regional nodal status.

### 3.4. 5-Fu and Paclitaxel Treatment

**Figure 3:**
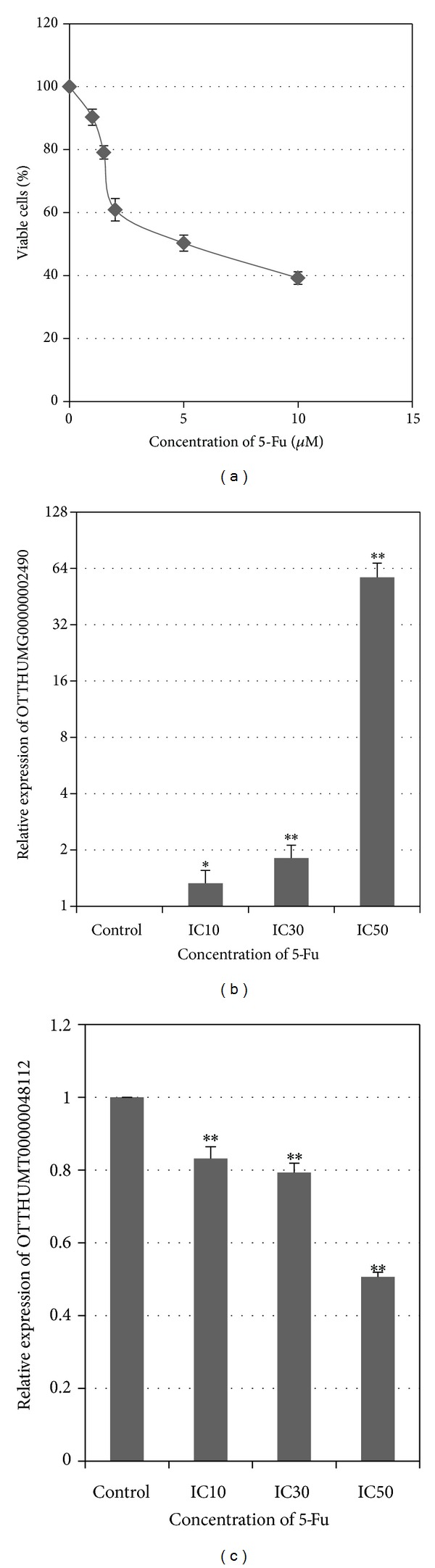
Effects of 5-Fu treatment on the expression of lncRNA in HN21B cells. (a) Dose-response curve of HN21B upon 5-Fu treatment. (b) and (c) Effect of 5-Fu treatment on the expression of OTTHUMG00000002490 and OTTHUMT00000048112, respectively. Bar, SD; *n* = 3; *P < 0.05 and **P < 0.01 by *t*-test.

**Figure 4:**
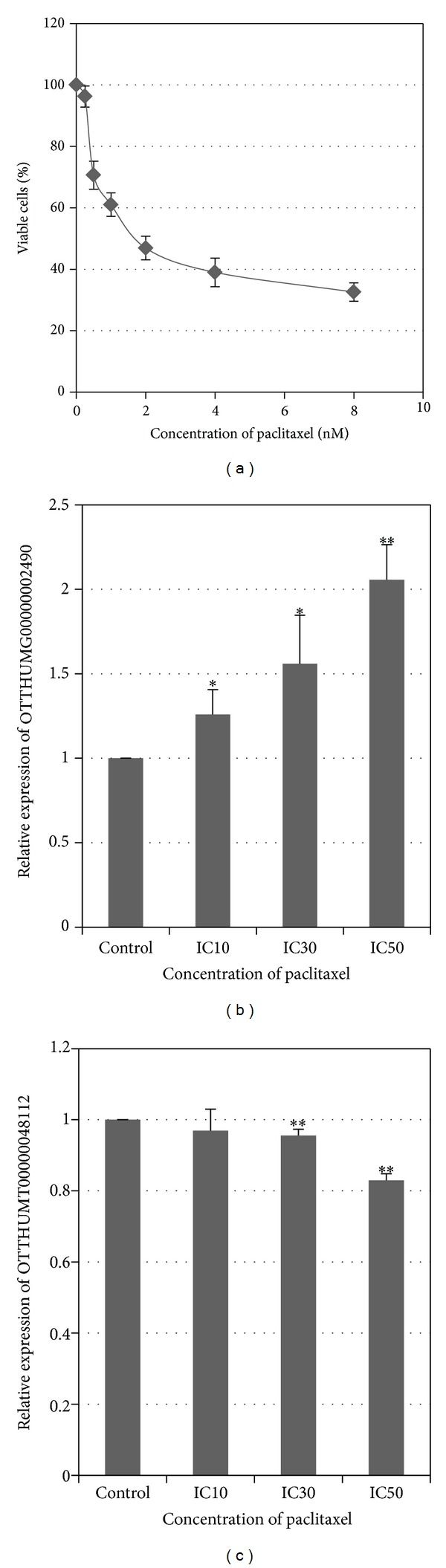
Effects of paclitaxel treatment on the expression of lncRNA in HN21B cells. (a) Dose-response curve of HN21B upon paclitaxel treatment. (b) and (c) Effect of paclitaxel treatment on the expression of OTTHUMG00000002490 and OTTHUMT00000048112, respectively. Bar, SD; *n* = 3; *P < 0.05 and **P < 0.01 by *t*-test.

To explore the potential functional role of the 2 identified lncRNAs with close correlation with the clinical features of the tongue SCC patients, we treated the cancer cell HN21B with 5-Fu and paclitaxel and examined the subsequent changes of lnc-MBL2-4 : 3 (OTTHUMT00000048112) and lnc-AL355149.1-1 (OTTHUMG00000002490) in response to the drug treatment. We use toxicity test to determine the inhibitory concentrations of 5-Fu and paclitaxel on tongue SCC cell line HN21B. For 5-Fu, the IC_10_, IC_30_, and IC_50_ were 0.6 *μ*M, 2.2 *μ*M, and 4.3 *μ*M, respectively (Figure 3(a)). 5-Fu treatment enhanced the expression of lnc-AL355149.1-1 (OTTHUMG00000002490), while it suppressed the expression of lnc-MBL2-4:3 (OTTHUMT00000048112) in a dose-dependent manner (Figure 3); for paclitaxel, the IC_10_, IC_30_, and IC_50_ on HN21B were 0.1 nM, 1.0 nM, and 2.2 nM, respectively (Figure 4(a)). Similar to the 5-Fu treatment, the expression of lnc-AL355149.1-1 (OTTHUMG00000002490) was activated upon paclitaxel treatment (Figure 4(b)). In addition, the expression of lnc-MBL2-4:3 (OTTHUMT00000048112) was suppressed under paclitaxel treatment in a dose-dependent manner (Figure 4(c)).

### 3.5. Expression Levels of MBL2-4:3 (OTTHUMT00000048112) and lnc-AL355149.1-1 (OTTHUMG00000002490) in the Cisplatin-Resistant HN21B Cells

**Figure 5:**
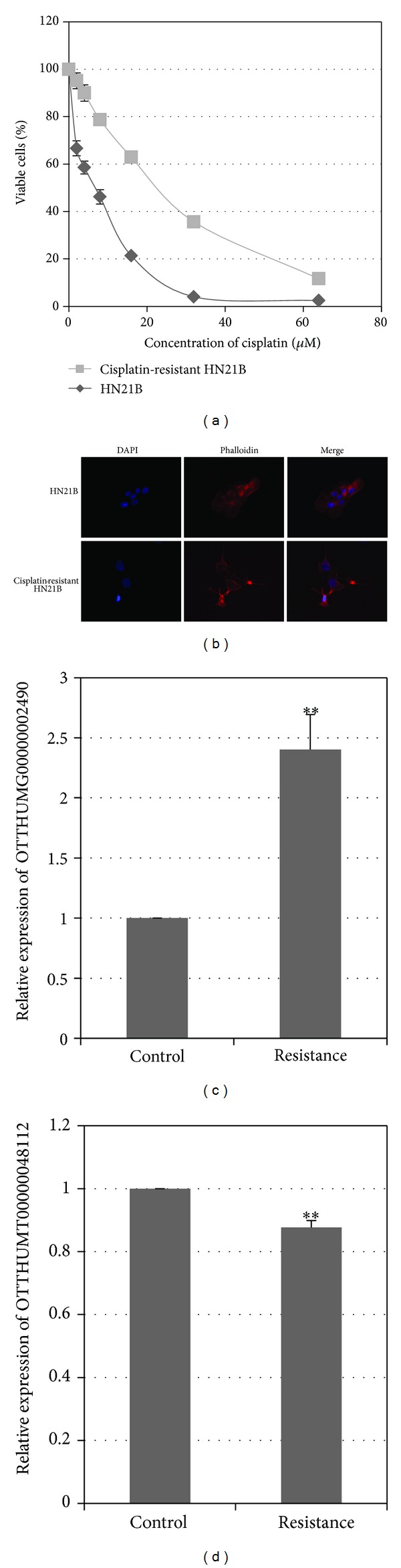
Differentially expressed lncRNA in cisplatin-resistant HN21B cells. (a) Dose-response curve of HN21B and cisplatin-resistant HN21B cells upon cisplatin treatment. (b) Morphology of HN21B and cisplatin-resistant HN21B cells. The nucleus was stained by blue-fluorescent DAPI; F-actin was labeled in red with Alexa Fluor 635 phalloidin. (c) and (d) Expression of OTTHUMG00000002490 and OTTHUMT00000048112 in HN21B and cisplatin-resistant HN21B cells. Bar, SD; *n* = 3; **P < 0.01 by *t*-test.

The cisplatin-resistant HN21B cells were developed by chronic treatment of HN21B cells with increasing concentration of cisplatin (Figure 5(a)). The IC_50_ of cisplatin-resistant HN21B was 22.7 *μ*M, which was obviously higher than that of the parental HN21B cells (7.0 *μ*M). Cisplatin-resistant HN21B cells displayed spindle-like changes in cell morphology, implicating a more aggressive phenotype (Figure 5(b)). Further, the cisplatin-resistant HN21B cells exhibited enhanced expression of lnc-AL355149.1-1 (OTTHUMG00000002490) and reduced expression of lnc-MBL2-4:3 (OTTHUMT00000048112) in comparison with parental HN21B cells (Figure 5).

## 4. Discussion

Expression alterations of lncRNA have been reported in several human tumors. In the past, lncRNA was regarded as functionless RNA fragment as it carries no protein coding information. With the characterization of functional non-coding RNA such as microRNA and PIWI-interacting RNAs (piRNAs) in the recent years, considerable attention has been dedicated to identify the active players during the progression and development of human malignancies. Although the deregulated lncRNA patterns have been identified in several cancers, such as prostate and liver cancers, little is known in oral tongue carcinoma. Using serial analysis of gene expression (SAGE), lncRNA expression has been demonstrated in the oral cavity and premalignant oral lesions located on tongue, gingiva, and buccal mucosa [11]. Recently, Fang et al. evaluated the expression patterns of lncRNA UCA1 (urothelial cancer-associated 1) in tongue squamous cell carcinoma and revealed that high UCA1 expression was linked to the migratory ability of the epithelial cancer cells and regional lymph node metastasis [12]. Another example is lncRNA MEG3 (maternally expressed gene 3). MEG3 is a lncRNA with the ability to regulate DNA methyltransferase 3B and was found to be downregulated in the tongue SCC tissues [13]. Low MEG3 level was an independent prognostic indicator and was associated with poor survival of tongue SCC patients [13]. The differentially expressed lncRNA could also be detected in the saliva and was suggested to be a useful noninvasive biomarker for oral cancer detection [14].

In this study, we used the existing microarray data to explore the differentially expressed lncRNA patterns in the tongue SCC tissues. The probesets on the microarray chips were originally designed to detect the protein-coding genes. Usually, multiple probes are assigned to individual genes to cover the entire length of the transcript in order to ensure the accuracy of measurement. With the advance in our understanding of lncRNA and their sequence, it was recognized that particular probesets on the microarray chips match with the lncRNA sequence. By reannotation of the microarray for lncRNA, the expression information of the microarray dataset could be revealed for subsequent inspection [15]. To ascertain that the identified lncRNA is deregulated in the tongue SCC, we carried out real-time quantitative PCR to validate the expression discrepancy between the epithelial cancers and normal epithelia of the same patients. One of the limitations of this study is the sample size of paired tumor and normal tissue is not big enough. Of the 8 lncRNAs, 4/8 (50%) were found to be deregulated in our tongue SCC cohort. To explore the potential role of the lncRNAs in the pathogenesis of tongue SCC, we correlated the expression levels with the clinicopathological parameters of tongue SCC patients. The association of lncRNA with T-stages and regional nodal status suggested that the lncRNA deregulation is potentially implicated in the rapid growth and high migratory property of the tongue SCC.

LncRNA could contribute to the cancer progression by controlling the process of proliferation and migration [16, 17]. In view of the association with tumor stages (OTTHUMG00000002490) and regional nodal status (OTTHUMT00000048112) of oral tongue patients, we suggested that lncRNA expression could possibly link to the progression of tongue squamous cell carcinoma. To explore the association, we treated the tongue cancer cell line with chemotherapeutic drugs which target highly proliferating cell and cell with high migratory potency [18, 19]. Both OTTHUMG00000002490 and OTTHUMT0000004811 demonstrated significant changes and the expression changes were responsive to the treatment dosage of 5-Fu and paclitaxel, revealing that the 2 lncRNAs can modulate the response of tongue cancer cells to chemotherapeutic agents. In addition, targeting lncRNA is suggested to be a feasible approach to overcome the resistance against chemotherapeutic drugs due to the observation that cancer cells expressing particular lncRNA are less responsive to the cytotoxic drugs [20, 21]. For example, in non-small cell lung cancer, it has been reported that cancer cells with high lncRNA AK126698 expression are associated with the cisplatin resistant phenotype [22]. As cisplatin-resistance is a challenge to the treatment efficacy of tongue squamous cell carcinoma, we developed a cisplatin-resistant model to examine the potential implication of the 2 lncRNAs to the development of resistant phenotype in oral tongue cancers. The significant changes in OTTHUMG00000002490 and OTTHUMT0000004811 observed in the resistant cell line indicated the potential association with the development of cisplatin resistance in tongue SCC.

## 5. Conclusions

In conclusion, our results indicated that oncogenic/tumor-suppressing lncRNA could potentially be identified through computational exploration of the existing microarray data. The associations of lncRNA with the clinical features of tongue SCC patients substantiate the claim that lncRNA deregulation is linked to the biology activity of tongue SCC. Whether the deregulated lnRNA could possibly be used as diagnostic/prognostic indicators has yet to be elucidated. Further studies are warranted to decipher the functional roles of lncRNA in the pathogenesis of tongue SCC.

## Figures and Tables

**Table 1:** Primer and probe sequences for qPCR.

Vega ID	LNCipedia ID	Forward primer (5′-3′)	Reverse primer (5′-3′)	UPL number∗
OTTHUMG00000020778	lnc-PPP2R4-5	tggattttcatgcctgctg	ggctgcattaccagaaaggt	3
OTTHUMG00000012449	lnc-SPRR2D-1	gcctctcctgcaagtgtga	tcctcatttatgacattttcagtctc	5
OTTHUMG00000012147	lnc-MAN1A2-1	gagaccgaggaatcttgctg	ctcagtgggctcagtaatgct	21
OTTHUMG00000015099	lnc-FAM46A-1	aggggtctcttgtccttggt	atcctcttattggcacactgc	26
OTTHUMT00000048111	lnc-MBL2-4:1	gcagccctggagagtttatct	cagcataatatggatgtttgaagg	67
OTTHUMT00000048112	lnc-MBL2-4:3	gagccagcaaaggagactga	cccagaaggggctcttactc	36
OTTHUMG00000002490	lnc-AL355149.1-1	gaaaactaggcgtctgggaac	caaacaatgggagcaagtcc	25
OTTHUMG00000015764	lnc-STXBP5-1	gctatgggaattatttttcctgtg	ggtaagccagttttcccttttt	16

*Probe number in the universal probe library.

**Table 2:** Differentially expressed lnRNA in tongue SCC microarray dataset identified by ncFANs reannotation.

Vega ID	LNCipedia ID	Locus conservation∗	Exon number	Transcript size (bp)	Genome location	Expression	*P* value
OTTHUMG00000020778	lnc-PPP2R4-5	Zebrafish	4	901	chr9: 132096185–132109678/+	Upregulated	0.007
OTTHUMG00000012449	lnc-SPRR2D-1	Mouse	2	1997	chr1: 152902516–152921686/−	Upregulated	0.019
OTTHUMG00000012147	lnc-MAN1A2-1	Mouse	3	511	chr1: 117838142–117863958/+	Upregulated	0.024
OTTHUMG00000015099	lnc-FAM46A-1	Mouse, zebrafish	2	520	chr6: 82523003–82523874/−	Upregulated	0.037
OTTHUMT00000048111	lnc-MBL2-4:1	Mouse	4	570	chr10: 54210637–54230293/−	Upregulated	0.049
OTTHUMT00000048112	lnc-MBL2-4:3	Mouse	3	975	chr10: 54210637–54230293/−	Upregulated	0.049
OTTHUMG00000002490	lnc-AL355149.1-1	No	2	327	chr1: 16847189–16848303/+	Downregulated	0.003
OTTHUMG00000015764	lnc-STXBP5-1	Mouse, zebrafish	2	1477	chr6: 147708800–147711601/+	Downregulated	0.006

*Locus conservation in *Mus musculus* and *Danio rerio* in comparison with *Homo sapiens*.

**Table 3:** Association of lncRNA expression levels with the clinicopathological variables in patients with tongue SCC.

		OTTHUMG00000020778	OTTHUMG00000012449	OTTHUMT00000048112	OTTHUMG00000002490
Gender					
Male	18	0.866	0.206	0.338	0.099
Female	14				
Age					
<55	16	0.270	0.254	0.809	0.669
>55	16				
T-stage					
T1-2	15	0.246	0.153	0.766	<0.001∗
T3-4	17				
Nodal stage					
Negative	18	0.925	0.065	0.002∗	0.145
Positive	14				
Smoker					
Yes	17	0.766	0.628	0.295	0.526
No	15				
Drinker					
Yes	12	0.552	0.477	0.387	0.387
No	20				

*P value below 0.05 was considered as statistical significance.
